# A Multi-Object Tracking Method with an Unscented Kalman Filter on a Lie Group Manifold

**DOI:** 10.3390/e28010103

**Published:** 2026-01-15

**Authors:** Xinyu Wang, Li Liu, Fanzhang Li

**Affiliations:** School of Computer Science and Technology, Soochow University, Suzhou 215000, China

**Keywords:** multi-object tracking, Kalman filter, Lie group, data association, motion model

## Abstract

Multi-object tracking (MOT) has attracted increasing attention and achieved remarkable progress. However, accurately tracking objects with homogeneous appearance, heterogeneous motion, and heavy occlusion remains a challenge because of two problems: (1) missing association due to recognizing an object as background and (2) false prediction caused by the predominant utilization of linear motion models and the insufficient discriminability of object appearance representations. To address these challenges, this paper proposes a lightweight, generic, and appearance-independent MOT method with an unscented Kalman filter (UKF) on a Lie group called LUKF-Track. The method utilizes detection boxes across the entire range of scores in data association and matches objects across frames by employing a motion model, where the propagation and prediction of object states are formulated using a UKF on the Lie group. LUKF-Track achieves state-of-the-art results on three public benchmarks, MOT17, MOT20, and DanceTrack, which are characterized by highly nonlinear object motion and severe occlusions.

## 1. Introduction

Multi-object tracking (MOT) involves detecting and tracking multiple objects within a video and is always a research hotspot in computer vision. MOT has a wide range of applications in scenarios including surveillance systems [1], self-driving vehicles [2], sports [3], behavior analysis [4], and augmented reality [5], to name but a few. However, MOT can be very challenging because maintaining the identities of multiple objects in complex environments requires handling various problems such as occlusions, motion blurs, interactions between objects, and changes in object appearances.

MOT methods fall into either tracking-by-detection (TBD) or tracking-free detection (TFD). For a long time, TBD has prevailed over TFD by virtue of its fewer interaction requirements, greater ability to identify randomly appearing objects in the middle of videos, and better performance in coping with various appearance distributions. Based on association metrics, MOT methods under the TBD framework can be broadly categorized as follows: motion-based (IoU matching, Kalman filter (KF)) [6,7,8], appearance-based (ReID embedding) [9,10,11], or a combination of both [12,13,14]. The motion model is used to predict the states and locations of objects based on their current observations, while the appearance model represents objects as fine-grained feature maps extracted from regions of detections and predictions. Using the calculated affinities between each pair of objects across frames, a matching strategy assigns identities to objects by the Hungarian algorithm [15] or greedy assignment [16,17].

Despite the advancements in MOT, tracking objects with homogeneous appearance, heterogeneous motion, and heavy occlusion remains a challenge because of the problems of missing association and false prediction. Missing association, also known as false negatives, refers to recognizing an object as background, which is usually caused by occlusion and low resolution. False prediction is often caused by the predominant utilization of linear motion models and the insufficient discriminability of appearance representations. For one, current motion models often apply a Kalman filter for motion prediction. However, the classical Kalman filter is designed for linear estimation systems, and this inherent limitation becomes evident when a motion model is confronted with complex, nonlinear motions and occlusions prevalent in dynamic environments, such as sports and dance. Additionally, most appearance models are sensitive to large rotations and drastic illumination changes, not to mention the tremendous cost in task-specific training for supervised learning methods.

Targeting the challenges in MOT, this paper proposes an MOT method with an unscented Kalman filter (UKF) on the Lie group referred to as LUKF-Track. The method makes full use of detection boxes from high scores to low ones in a two-round association process and matches objects across frames by using a motion model where the propagation and prediction of object states are formulated by a UKF on the Lie group. The Lie group provides geometric representations of continuous transformation through manifold structures and offers algebraic solutions to the modeled problems on manifold spaces, which improves the accuracy of prediction and anti-interference performance for complex, nonlinear motions and occlusions prevalent in dynamic environments. LUKF-Track was validated against three benchmark datasets and compared with other trackers. The results demonstrate that LUKF-Track is particularly effective in scenarios characterized by highly nonlinear motion and severe occlusion. The primary contribution of this paper is twofold, as follows:

(1) We propose a generic and appearance-independent MOT method that pushes forward the state-of-the-art tracking performance in three MOT experiments.

(2) The locations and propagations of objects are represented as a motion model formulated by a UKF on the Lie group, which provides additional insight into the dynamics of objects and thus effectively solves the false prediction problem for tracking under occlusion and nonlinear motion.

## 2. Related Works

### 2.1. Multiple Object Tracking

In MOT, the current leading paradigm is TBD, which detects objects in each individual frame and subsequently associates the detections with the tracking process over time. The process of associating data for target tracking typically encompasses two fundamental components: the calculation of object similarity and the implementation of a matching strategy. The calculation of similarity is dependent upon the detection results (e.g., the boundary boxes and center coordinates of the objects) as the input and the calculation of the similarity between the detection target and the tracking target. This can be performed by defining an association metric of objects with either one or a combination of motion and appearance models.

**Motion model.** The motion model defines an association metric based on motion consistency and often involves corresponding measured states of detection to predicted states by using a variant of the Kalman filter. Typical trackers that use the motion model alone for data association include SORT [18], ByteTrack [6], and UCMCTrack [8]. SORT uses a standard Kalman filter to predict the location of the detected targets with high scores, then computes the IoU between detection boxes and predicted boxes as affinity. ByteTrack tracks by associating almost every detection box instead of only the high-score ones. For low-score detection boxes, the method utilizes their similarities with tracklets to recover true objects and filter out the background [6]. UCMCTrack replaces the IoU with the Mapped Mahalanobis Distance to calculate the affinity between objects by projecting the probability distribution onto the ground plane [8].

**Appearance model.** The appearance model defines an association metric based on the appearance similarity between object-level features. Different from the motion model, which mainly focuses on short-range matching, the appearance model is more helpful in long-range matching. Typical trackers that use an appearance model alone for data association include UniTrack [9], FineTrack [10], and the self-supervised learning appearance model [11]. UniTrack consists of a single and task-agnostic appearance model, which can be learned in a supervised or self-supervised fashion, and multiple “heads” that address individual tasks and do not require training [9]. FineTrack utilizes diverse local embeddings and background-filtered global embeddings to jointly describe appearance [10]. The self-supervised learning appearance model leverages Momentum Contrastive Learning (MoCo-v2) [19] to learn an appearance representation embedding model and extract features from detection without using tracking annotations [11].

**A combination of both.** A lot of trackers combine motion and appearance information into a more informed association metric. Examples include DeepSORT [20], BoT-SORT [12], and Rt-Track [14]. DeepSORT combines appearance information generated by a CNN-based deep appearance descriptor with motion information computed with a Kalman filter, which increases robustness against misses and occlusions while keeping the system easy to implement, efficient, and applicable to online scenarios [20]. BoT-SORT incorporates the re-identification (ReID) of objects by deep appearance cues and camera motion compensation to the framework of ByteTrack [12]. Rt-Track combines the direction consistency generated from a trajectory smoothing mechanism and the appearance features obtained by an ultra-granular feature extraction network to a similarity metric [14].

The proposed LUKF-Track associates objects predominantly based on their motion consistency and uses appearance similarity as an alternative option for some long-range association tasks.

### 2.2. The Application of Lie Group Machine Learning in Computer Vision

Lie group machine learning is an interdisciplinary research field combining Lie group theory with machine learning methods. Using the structural characteristics of Lie groups, Lie group machine learning has a unique advantage in processing data with transformation invariance. Since the early 2000s, a series of Lie group machine learning algorithms have been designed, including Lie group meta learning [21,22], Lie group semi-supervised learning [23,24], Lie group kernel learning [25], Lie group transfer learning [26], etc., and they have been used to solve problems in pattern recognition, robotics, computer vision, and natural language processing. Specifically in computer vision, Lie group machine learning finds its success in a variety of applications, such as object tracking [27], pose estimation [28], action representation and recognition [29,30], simultaneous localization and mapping (SLAM) [31], motion identification [32], and so on. In [27], an object tracking algorithm is presented based on an unscented particle filter for systems where the states of objects evolve on a Lie group space. In [28], a filter on the Lie group is proposed for pose estimation, where the time propagation of the state is formulated on the Lie algebra. To address the complexity and nonlinearity of human action recognition in videos, Lie group features are combined with deep learning to provide a natural representation of complex and diverse action data [29]. Wang et al. [30] develop a hand-worn prototype device for gesture recognition by using Lie group theory to capture the inherent structural changes in hand movements and the spatiotemporal dependencies between multiple bones. Labsir et al. [31] address the problem of monocular SLAM by estimating both the state and the map on Lie group space through a Lie group-based extended Kalman filter. Kachhoria et al. [32] acquire highly suitable Lie grouping characteristics, which enable 3D motion identification by integrating the Lie group topology into a deep networking design.

## 3. Proposed Method

### 3.1. System Modeling

**Figure 1:**
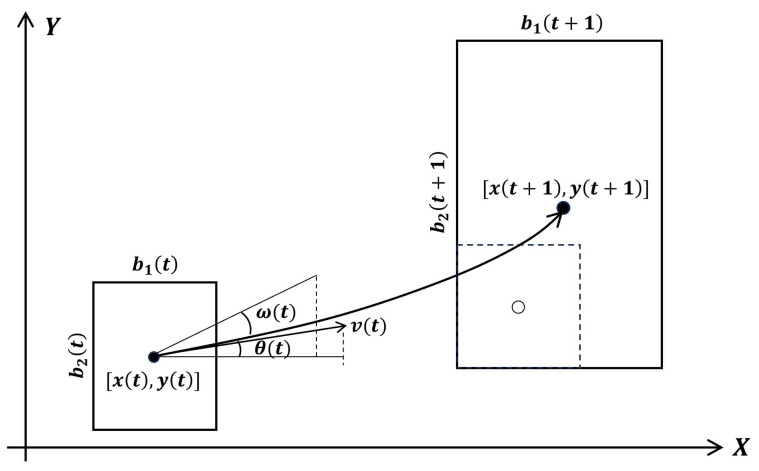
Motion model and parameters of target detection in neighboring video frames.

The motion of a detection box across frames can be modeled as a target performing a trajectory with constant speed and angular rate in a plane. Figure 1 illustrates the motion of a detection box in two consecutive frames along with the parameters used for system modeling. As shown in the figure, *x*, *y*, $b_{1}$, $b_{2}$, θ, υ, and ω denote the state parameters of a detection box, corresponding to the x-position, y-position, width, height, heading angle, translational speed, and angular rate. Using triangle similarity and basic trigonometry, the relationships among these parameters can be approximately expressed as follows:

(1) $$\frac{x\left(t+1\right)-x\left(t\right)}{\upsilon \left(t\right)}\approx \left[\frac{\sin\left(\theta \left(t\right)+\omega \left(t\right)\right)-\sin\theta \left(t\right)}{\omega \left(t\right)}\right]$$

(2) $$\frac{y\left(t+1\right)-y\left(t\right)}{\upsilon \left(t\right)}\approx \left[\frac{\cos\theta \left(t\right)-\cos\left(\theta \left(t\right)+\omega \left(t\right)\right)}{\omega \left(t\right)}\right]$$

Considering the system noises in the motion, the final position of a detection box will not be deterministic, and the two expressions above can be further expanded to an equation set modeling the dynamics of the system:

(3) $$\begin{array}{cc} x\left(t+1\right) & =x\left(t\right)+\upsilon \left(t\right)\left[\cos\theta \left(t\right)\frac{\sin\omega \left(t\right)}{\omega \left(t\right)}-\sin\theta \left(t\right)\frac{1-\cos\omega \left(t\right)}{\omega \left(t\right)}\right]+q_{1}\left(t\right)\\ y\left(t+1\right) & =y\left(t\right)+\upsilon \left(t\right)\left[\cos\theta \left(t\right)\frac{1-\cos\omega \left(t\right)}{\omega \left(t\right)}+\sin\theta \left(t\right)\frac{\sin\omega \left(t\right)}{\omega \left(t\right)}\right]+q_{2}\left(t\right)\\ \theta \left(t+1\right) & =\theta \left(t\right)+\omega \left(t\right)+q_{3}\left(t\right)\\ \upsilon \left(t+1\right) & =\upsilon \left(t\right)+q_{4}\left(t\right)\\ \omega \left(t+1\right) & =\omega \left(t\right)+q_{5}\left(t\right) \end{array}$$

where the vector $q\left(t\right)=\left[q_{1}\left(t\right),q_{2}\left(t\right),q_{3}\left(t\right),q_{4}\left(t\right),q_{5}\left(t\right)\right]∼N_{R^{5}}\left(0,P_{qq}\right)$ is a white Gaussian noise in $R^{5}$. According to [33], the pose of a moving object presents a banana-shaped distribution, and the matrix Lie Special Euclidean group $SE\left(2\right)$ provides a better means of handling the uncertainty distributions than in Euclidean space. Assuming that a detection box with dynamics modeled as Equation (3) presents a banana-shaped distribution, its pose can be represented by an $SE\left(2\right)$ Lie group. The translational speed and angular rate are expressed in $R^{2}$. The resulting group is a 5-dimensional matrix Lie group *G* obtained by the Cartesian product of $SE\left(2\right)$ and $R^{2}$, i.e., $G=SE\left(2\right)\times R^{2}$. The state can be estimated by a group element *g* on $G:g∼N_{R^{6}}\left(\bar{g},P_{gg}\right)$ and is written as a matrix form:

(4) $$g\left(t\right)=\left[\begin{array}{cccccc} \cos\theta (t) & -\sin\theta (t) & x(t) & \; & & \\ \sin\theta (t) & \cos\theta (t) & y(t) & \; & 0_{3\times 3}\\ 0 & 0 & 1 & \; & & \\ \; & \; & & 1 & 0 & v(t)\\ \; & 0_{3\times 3} & \; & 0 & 1 & \omega (t)\\ \; & \; & & 0 & 0 & 1 \end{array}\right]$$

Accordingly, the system dynamics described by (3) can be estimated by *g* written as follows:

(5) $$g\left(t+1\right)=g\left(t\right)\exp_{G}\left(\Omega \left(g\left(t\right)\right)+q\left(t\right)\right)$$

where $\Omega :G\to R^{5}$ is a nonlinear $C^{2}$ function. Note that although $g\left(t\right)$ is a 6×6 matrix, *G* is a 5-dimensional Lie group. This happens because we employ 3×3 matrices to represent $R^{2}$, a two-dimensional space on the matrix Lie group. $g\left(t\right)$ is assumed to be a concentrated Gaussian distribution on *G*: $g\left(t\right)∼N_{G}\left(\bar{g}\left(t\right),P_{gg}\right)$. The function $\Omega \left(g\left(t\right)\right)∼N_{R^{5}}\left(0,P\left(t|t\right)\right)$ captures the dynamics of the system, and it can be obtained from a formula manipulation to Equation (5):

(6) $$\Omega \left(g\left(t\right)\right)=\log_{G}\left[g\left(t+1\right)g^{-1}\left(t\right)\right]-q\left(t\right)$$

where the logarithm map of $g\left(t\right)$ is a Lie algebra associated with the matrix Lie group *G*:

(7) $$\log_{G}\left[g\left(t\right)\right]=\left[\begin{array}{cccccc} 0 & -\theta (t) & p_{x}\left(t\right) & \; & & \\ \theta (t) & 0 & p_{y}\left(t\right) & \; & 0_{3\times 3}\\ 0 & 0 & 0 & \; & & \\ \; & \; & & 0 & 0 & v(t)\\ \; & 0_{3\times 3} & \; & 0 & 0 & \omega (t)\\ \; & \; & & 0 & 0 & 0 \end{array}\right]$$

with

(8) $$\left[\begin{array}{c} p_{x}\left(t\right)\\ p_{y}\left(t\right) \end{array}\right]=\frac{\theta (t)}{2(1-\cos\theta (t))}\left[\begin{array}{cc} \sin\theta (t) & 1-\cos\theta (t)\\ \cos\theta (t)-1 & \sin\theta (t) \end{array}\right]\left[\begin{array}{c} x(t)\\ y(t) \end{array}\right]$$

### 3.2. Measurement Modeling

**Figure 2:**
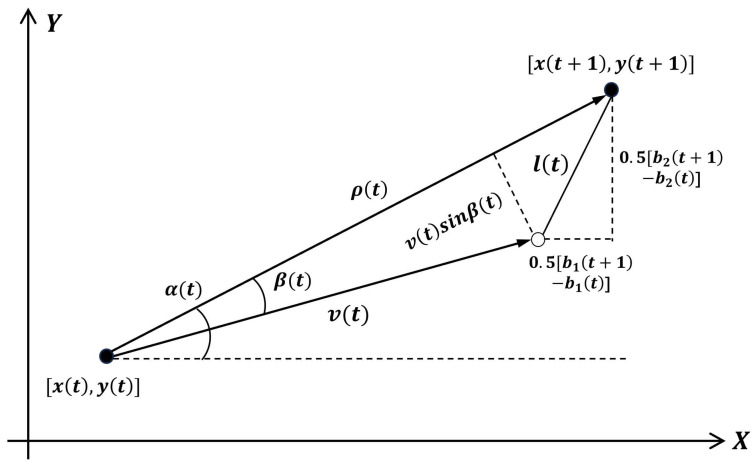
Measurement model and parameters of target detection in neighboring video frames.

We consider three measurements from a detection: azimuth $\alpha \left(t\right)$, range $\rho \left(t\right)$, and radial speed $\beta \left(t\right)$. As shown in Figure 2, the measurements at timestep *t* can be calculated from the state parameters once the detection box reaches timestep t+1. Using triangle similarity and simple trigonometry, the relations between these parameters can be expressed as follows:

(9) $$\mathrm{tg}\left(\alpha \left(t\right)\right)=\frac{y\left(t+1\right)-y\left(t\right)}{x\left(t+1\right)-x\left(t\right)}$$

(10) $$4l^{2}\left(t\right)={\left[b_{2}\left(t+1\right)-b_{2}\left(t\right)\right]}^{2}+{\left[b_{1}\left(t+1\right)-b_{1}\left(t\right)\right]}^{2}$$

(11) $$\begin{array}{cc} \rho \left(t\right) & =v\left(t\right)\cos\beta \left(t\right)+\sqrt{l^{2}\left(t\right)-{\left(v\left(t\right)\sin\beta \left(t\right)\right)}^{2}} \end{array}$$

Considering the noise in measuring the motion as a white Gaussian noise $s\left(t\right)=\left[s_{1}\left(t\right),s_{2}\left(t\right),s_{3}\left(t\right)\right]∼N_{R^{3}}\left(0,P_{ss}\right)$, the measurements of a detection box at timestep *t* can be represented as the following function set:

(12) $$\begin{array}{cc} \alpha (t) & =\arctan\frac{y(t+1)-y(t)}{x(t+1)-x(t)}+s_{1}\left(t\right)\\ \rho (t) & =\sqrt{{\left[x(t+1)-x(t)\right]}^{2}+{\left[y(t+1)-y(t)\right]}^{2}}+s_{2}\left(t\right)\\ \beta (t) & =\arccos\frac{\rho^{2}\left(t\right)+v^{2}\left(t\right)-l^{2}\left(t\right)}{2\rho (t)v(t)}+s_{3}\left(t\right) \end{array}$$

The measurements arrive in polar coordinates and can be written in the structure of the Lie group $H:h\left(t\right)=\exp_{H}\left[\tilde{\alpha }\left(t\right),\tilde{\rho }\left(t\right),\tilde{\beta }\left(t\right)\right]$. As stated in [33], the distributions of measurements also resemble banana-shaped contours. Because of this, the chosen Lie group for the measurements will be constructed as $H=SO\left(2\right)\times R^{2}$. Thus, the measurement map Φ:G→H is given as follows:

(13) $$\Phi \left[g(t)\right]=\left[\begin{array}{ccccc} \cos\alpha (t) & -\sin\alpha (t) & \; & 0_{2\times 3} & \\ \sin\alpha (t) & \cos\alpha (t) & \; & & \\ \; & \; & 1 & 0 & \rho (t)\\ 0_{3\times 2} & \; & 0 & 1 & \beta (t)\\ \; & \; & 0 & 0 & 1 \end{array}\right]$$

Combining Equations (12) and (13), the dynamics of measurement can be modeled on the Lie group.

(14) $$h\left(t\right)=\Phi \left(g\left(t\right)\right)\exp_{H}\left(s\left(t\right)\right)$$

### 3.3. State Prediction

It is assumed that the posterior state distribution after the arrival of the measurement is a concentrated Gaussian distribution on Lie groups, i.e., $g\left(t|t\right)∼N_{G}\left(g\left(t|t\right),P\left(t|t\right)\right)$. At each timestep t>0, three sigma points $G^{\left(i\right)}\left(t|t\right)∼N_{G}\left(0,P\left(t|t\right)\right)$, $Q^{\left(i\right)}\left(t|t\right)∼N_{R^{5}}\left(0,P_{qq}\right)$, and $S^{\left(i\right)}\left(t|t\right)$ $∼N_{R^{3}}\left(0,P_{ss}\right)$ along with their weights are created for $g\left(t\right)$, $q\left(t\right)$, and $s\left(t\right)$ according to Equations (1) and (2). The computed $G^{\left(i\right)}\left(t|t\right)$ is propagated through the system model in Equation (5) and can be calculated as follows:

(15) $$G^{\left(i\right)}\left(t+1|t\right)=G^{\left(i\right)}\left(t|t\right)\exp_{G}\left[\Omega \left(G^{\left(i\right)}\left(t|t\right)\right)+Q^{\left(i\right)}\left(t|t\right)\right]$$

Based on the propagation of the sigma points $G^{\left(i\right)}$ in Equation (15), the predicted state $g\left(t+1|t\right)∼N_{G}\left(g\left(t+1|t\right),P\left(t+1|t\right)\right)$ along with its covariance can be calculated. It is noted that $g\left(t\right)$ is a concentrated Gaussian distribution on *G* and can be written as $g\left(t\right)=\bar{g}\left(t\right)\exp_{G}\left(\epsilon \left(t\right)\right)$ or more directly $g\left(t\right){\bar{g}}^{-1}=\exp_{G}\left(\epsilon \left(t\right)\right)$ with $\epsilon \left(t\right)∼N_{R^{5}}\left(0,P_{gg}\right)$. To make sure the state prediction $g\left(t+1|t\right)$ is also a concentrated Gaussian distribution, we replaced the numerical mean with a Lie group intrinsic mean:

(16) $$g\left(t+1|t\right)=\exp_{G}\left[\sum_{i=0}^{11}W_{m}^{\left(i\right)}\log_{G}\left[G^{\left(i\right)}\left(t+1|t\right){\bar{g}}^{-1}\left(t+1|t\right)\right]\right]$$

The covariance calculation is modified accordingly:

(17) $$\begin{array}{cc} P\left(t+1|t\right)= & \sum_{i=0}^{11}W_{c}^{\left(i\right)}\log_{G}\left[G^{\left(i\right)}\left(t+1|t\right)g^{-1}\left(t+1|t\right)\right]\;\cdot \;\log_{G}{\left[G^{\left(i\right)}\left(t+1|t\right)g^{-1}\left(t+1|t\right)\right]}^{T} \end{array}$$

### 3.4. State Update

In the update step, we apply the UT to the measurement model in Equation (14) to approximate the measurement statistics and further update the system state at timestep t+1. Instead of creating a new set of sigma points for $g\left(t\right)$, the propagated sigma points $G^{\left(i\right)}\left(t+1|t\right)$ and the sigma points of $s\left(t\right)$ obtained in the previous section are used in calculating the propagation of the sigma points for $h\left(t\right)$:

(18) $$H^{\left(i\right)}\left(t+1|t\right)=\Phi \left(G^{\left(i\right)}\left(t+1|t\right)\right)+\exp_{H}\left[S^{\left(i\right)}\left(t|t\right)\right]$$

It is noted that $h\left(t\right)$ is a concentrated Gaussian distribution on *H* and can be written as $h\left(t\right)=\bar{h}\left(t\right)\exp_{H}\left(\delta \left(t\right)\right)$ with $\delta \left(t\right)∼N_{R^{3}}\left(0,P_{hh}\right)$. To make sure the propagation of the measurement $h\left(t+1|t\right)$ is also a concentrated Gaussian distribution, we compute the mean of the sigma point $H^{\left(i\right)}$ to update the state at the moment t+1:

(19) $$h\left(t+1|t\right)=\exp_{H}\left[\sum_{i=0}^{11}W_{m}^{\left(i\right)}\log_{H}\left[H^{\left(i\right)}\left(t+1|t\right){\bar{h}}^{-1}\left(t+1|t\right)\right]\right]$$

Accordingly, the covariance of the measurement and the cross-covariance between prediction and measurement can be modified as follows:

(20) $$\begin{array}{cc} P_{hh}= & \sum_{i=0}^{11}W_{c}^{\left(i\right)}\log_{H}\left[H^{\left(i\right)}\left(t+1|t\right)h^{-1}\left(t+1|t\right)\right]\;\cdot \;\log_{H}{\left[H^{\left(i\right)}\left(t+1|t\right)h^{-1}\left(t+1|t\right)\right]}^{T} \end{array}$$

(21) $$\begin{array}{cc} P_{gh}= & \sum_{i=0}^{11}W_{c}^{\left(i\right)}\log_{G}\left[G^{\left(i\right)}\left(t+1|t\right)g^{-1}\left(t+1|t\right)\right]\;\cdot \;\log_{H}{\left[H^{\left(i\right)}\left(t+1|t\right)h^{-1}\left(t+1|t\right)\right]}^{T} \end{array}$$

We cannot apply the filter update directly on $g\left(t\right)$ based on the measurement $h\left(t\right)$ because they are on different Lie group spaces; instead we update the random variable $\epsilon \left(t\right)∼N_{R^{5}}\left(0,P_{gg}\right)$ based on $\delta \left(t\right)∼N_{R^{3}}\left(0,P_{hh}\right)$, which is obtained from the arrived measurement $h\left(t+1\right)$:

(22) $$\delta \left(t+1\right)=\log_{H}\left[h\left(t+1\right)h^{-1}\left(t+1|t\right)\right]$$

(23) $$\begin{array}{cc} \epsilon \left(t+1|t+1\right) & =P_{gh}P_{hh}^{-1}\log_{H}\left[h\left(t+1\right)h^{-1}\left(t+1|t\right)\right] \end{array}$$

Finally, the prediction on state $g\left(t+1|t\right)$ and the covariance of the prediction $P\left(t+1|t\right)$ can be updated to $g\left(t+1|t+1\right)$ and $P\left(t+1|t+1\right)$:

(24) $$\begin{array}{cc} \; & g\left(t+1|t+1\right)=g\left(t+1|t\right)\exp_{G}\left[P_{gh}P_{hh}^{-1}\log_{H}\left(h\left(t+1\right)h^{-1}\left(t+1|t\right)\right)\right] \end{array}$$

(25) $$\begin{array}{cc} P\left(t+1|t+1\right) & =P\left(t+1|t\right)-P_{gh}P_{hh}^{-1}P_{gh}^{T} \end{array}$$

where $g\left(t+1|t+1\right)$ and $P\left(t+1|t+1\right)$ are the updated target state and variance at t+1.

### 3.5. Data Association

LUKF-Track applies a two-round association process to match pairs of objects across frames. After separating low-scored and high-scored detected objects, motion models based on LUKF are constructed for all tracks to predict new locations in the current frame.

The first association is performed between high-scored detections and all tracks, including the lost tracks from previous frames. The association metric can be computed by either the IoU or the Re-ID feature distances between detection and prediction. Considering that appearance information is helpful in the long-range association for preserving the identity of tracks, we use appearance similarity as an alternative association metric to re-identify a pair of objects separated by a period of time. Following the method in [25], an appearance feature of an object is constructed by calculating a matrix Lie group from a series of extracted image features: color, gradient, texture, and local context. The appearance similarity between two objects is measured by a geodesic distance between the corresponding matrix Lie groups $M_{1}$ and $M_{2}$:

(26) $$D_{LG}\left(M_{i},M_{j}\right)={∥log\left(M_{i}^{-1}M_{j}\right)∥}_{F}$$

where ${∥\;\cdot \;∥}_{F}$ is the Frobenius norm. Then, the Hungarian algorithm is used to finish the matching.

The second association is performed between low-scored detections and the remaining tracks after the first association. Here, the IoU is chosen because low-scored detections often contain severe occlusion or motion blur, and appearance features are not reliable. For an unmatched track after the second association, only when it exists for more than a certain number of frames, such as 30, do we delete it from the tracking array.

The pseudo-code of LUKF-Track is shown in Algorithm 1.


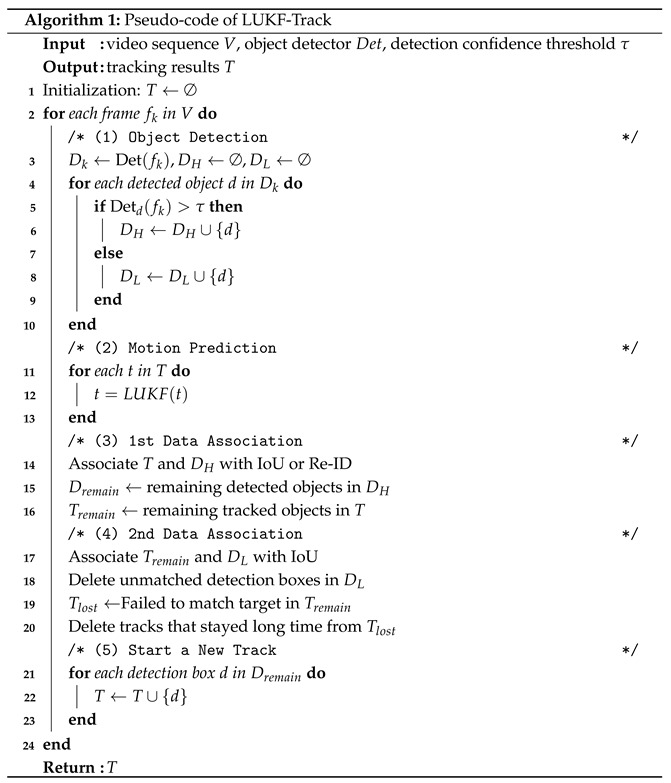


## 4. Experiments and Analysis

### 4.1. Experiment Setting

**Datasets.** The experimental data presented in this paper comprises three benchmark datasets for multi-target tracking: MOT17 [34], MOT20 [35], and DanceTrack [36]. MOT17 and MOT20 are about pedestrian tracking, where targets mostly move linearly, and scenes in MOT20 are more crowded. DanceTrack [54] features dancers (i.e., objects) with similar appearances, fast motion, and heavy occlusion.

**Methods and Metrics.** Seven MOT methods published in recent years were chosen for comparison: SORT [18], DeepSORT [20], StrongSORT [13], OC-SORT [7], QDTrack [37], FineTrack [10], and ByteTrack [6]. These methods can be further categorized into three groups by association metrics. The proposed LUKF-Track is implemented in two ways: one uses both motion and appearance information (i.e., LUKF-Track (M+A)); the other uses motion information only (i.e., LUKF-Track (M)). All methods are evaluated with three MOT metrics: HOTA [38] for higher-order tracking accuracy, MOTA [39] for multi-target tracking accuracy, and IDF1 for target recognition [40].

**Implementation.** For a fair comparison, we directly use the publicly available YOLOX [41] detector weights by ByteTrack. The detection score threshold τ is set to 0.6 for categorizing a detection as high- or low-confidence. The IoU threshold for matching is set to 0.2, which means that if the overlap between the detection box and prediction box is below 0.2, the matching calculation will not be triggered. A duration of 30 frames is set to prevent the missing of reappearing objects and filter out unreasonable association pairs.

### 4.2. Results and Discussion

The benchmark results of LUKF-Track and the chosen MOT trackers for comparison on three benchmark datasets are reported and compared. We then compare LUKF-Track (M) with ByteTrack in dealing with several typical challenging scenarios with visualization results. The reason why we chose ByteTrack instead of other trackers for further analysis is that LUFK-Track adopts the same weights of detection and a similar way of handling low-scored detections.

#### 4.2.1. Benchmark Results

Table 1 summarizes the test results of all eight MOT methods on the three datasets. For each performance metric, the highest score is highlighted in bold and underlined, and the second highest score is highlighted by an underline.

**MOT17.** The two implementations of LUKF-Track rank second and third and are only outperformed by FineTrack, which achieves the best score in HOTA and the second best scores in both MOTA and IDF1. Specifically, LUKF-Track (M+A) achieves the best IDF1 (79.6) and the second best HOTA (63.7) and ranks third in MOTA (78.5). Moreover, it outperforms the two trackers that use both motion and appearance information (i.e., DeepSort and StrongSort). LUKF-Track (M) presents the third best overall performance and prevails in HOTA (63.7 vs. 63.1) and IDF1 (79.5 vs. 77.3) compared with ByteTrack. The relatively low scores of LUKF-Track in MOTA (i.e., 78.2 and 78.5 for the two implementations) may be attributed to the high ID switches caused by a suboptimal IoU setting.

**MOT20.** LUKF-Track (M+A) outperforms all other trackers in MOT20, which is under severe pedestrian occlusion, setting the highest MOTA of 78.1 and IDF1 of 79.2, and an average score of HOTA (61.9). As the runner-up, FineTrack achieves the highest HOTA (63.6), the second highest MOTA (77.9), and the third highest IDF1 (79.0). LUKF-Track (M), which ranks third among all trackers, prevails in HOTA (62.2 vs. 61.3) and IDF1 (79.1 vs. 75.2) compared with ByteTrack.

**DanceTrack.** Overall, LUKF-Track (M) achieves the second best performance after FineTrack by presenting the second best scores in both MOTA (91.3) and IDF1 (56.1), which is better than LUKF-Track (M+A) and outperforms ByteTrack by a large margin: +1.8 MOTA, +2.5 HOTA, and +3.6 IDF1. However, both implementations of LUKF-Track present relatively low scores in HOTA (i.e., 49.4 and 49.8), which can be explained by the unbalances among accurate detection, association, and localization.

In general, both implementations of LUKF-Track achieve competitive tracking performances, especially in the experiments on MOT17 and MOT20. The results demonstrate that the proposed motion model is effective, as it accumulates fewer tracking errors and assigns IDs to objects more accurately. As LUKF-Track is designed to be simple for better generalization, we use the default detection confidence threshold and apply a shared parameter stack across the three datasets, which is different from other trackers such as FineTrack and StrongSort. We believe that carefully tuning the parameters for each dataset can further boost their performance.

#### 4.2.2. Visualization Results

**Figure 3:**
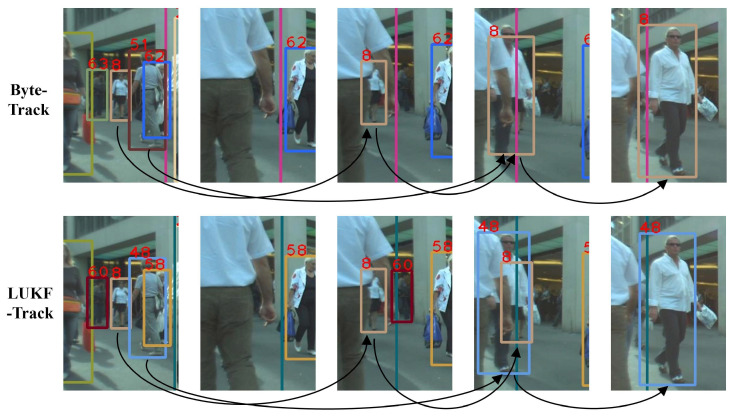
A scenario of object occlusion due to the appearance of a new pedestrian and changes in the object’s appearance across frames. ByteTrack (**the first row**) loses track by assigning the ID of the first pedestrian to a new pedestrian, while LUKF-Track (**the second row**) identifies the appearance of the new pedestrian.

**Figure 4:**
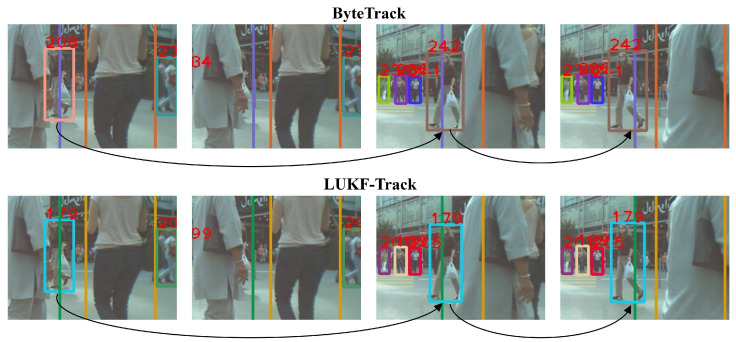
A scenario of object occlusion due to there being many pedestrians. ByteTrack (**the first row**) loses track by assigning a new ID to the pedestrian after their reappearance, while LUKF-Track (**the second row**) keeps the original ID of the pedestrian.

Figure 3 and Figure 4 present two chosen video sequences with tracking boxes to compare LUKF-Track (M) with ByteTrack when dealing with difficult cases in videos from MOT17, including partial occlusion, complete occlusion, changes in object appearance, and the appearance of new objects.

In Figure 3, a distant pedestrian (i.e., small object) in the orange box (the eighth in both ByteTrack and LUFK-Track) first approaches, then interacts with two pedestrians nearby. ByteTrack loses the tracking for the distant pedestrian in the fourth frame when the three people gather and assigns an ID of 8 to a person who blocks the first pedestrian, while LUFK-Track keeps the tracking on the distant pedestrian and assigns a new ID to the person nearby in the fourth frame.

In Figure 4, a pedestrian (208th in ByteTrack, 179th in LUKF-Track) is first partially occluded, then completely occluded, and finally reappears. After a temporary disappearance due to a complete occlusion in the second frame, ByteTrack reassigns an ID of 242 instead of the original 208 to the pedestrian in the third frame. In contrast, LUKF-Track is able to recognize the pedestrian and assigns the same ID to the person after her reappearance in the fourth frame.

**Figure 5:**
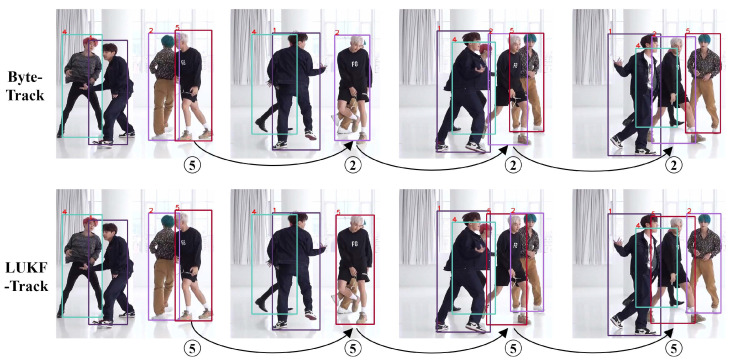
A scenario of a group dance with heavy dancer interactions and motion blurs. ByteTrack (**the first row**) loses the tracking on one dancer by assigning a new ID of 2 instead of the original 5, while LUKF-Track (**the second row**) preserves the original object ID.

Figure 5 presents a video sequence to compare LUKF-Track with ByteTrack in handling difficult cases in the videos of DanceTrack, including partial occlusion, complete occlusion, motion blur, interactions between objects, and changes in object appearance. The two dancers (second and fifth) in the first frame exchange positions from the second to the third frame, and due to the complete occlusion of the second frame, ByteTrack confuses the two dancers in the third frame, recognizing the dancer with the original ID of 2 as the dancer with the original ID of 5 and recognizing the dancer with the original ID of 5 as the dancer with the original ID of 2. While Lie-ByteTrac is able to recognize the dancer in the first frame and maintains the same ID after the two dancers interact with each other, the correct ID assignment is performed.

**Figure 6:**
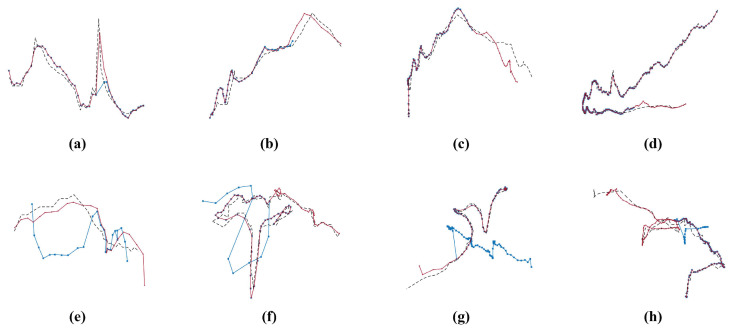
Eight selected tracking trajectories output by ByteTrack and LUKF-Track for the MOT17 dataset. The black dashed line, blue dot, and red line are used to represent the ground truth trajectory and tracking trajectories generated from ByteTrack and LUKF-Track.

We further compare LUKF-Track with ByteTrack by their trajectory visualizations for the MOT17 dataset. In Figure 6, the black dashed line, blue dot, and red line are used to represent the ground truth (GT) trajectory and tracking trajectories output by the two methods. Overall, the tracking trajectory of LUKF-Track is closer to the GT and presents fewer ID switches, fragment tracks, and object losses from which ByteTrack suffers. In Figure 6a, LUKF-Track accurately tracks the occurrence of an abrupt jump in motion, and its trajectory is close to that of the GT, while ByteTrack fails to track this drastic motion by responding with a relatively weak one. In Figure 6b–d, the tracking trajectory of ByteTrack becomes abnormal after a certain point. This happens when the ID of an object is wrongly assigned to some other object (i.e., ID switch). In contrast, LUKF-Track avoids the occurrence of this problem in these scenarios. In Figure 6e, although the trajectories of both trackers are steeper than that of the GT, the difference in LUKF-Track compared with the GT is smaller. In Figure 6f–h, the trajectory of ByteTrack vanishes after a certain point when the tracker loses track of the object, while LUKF-Track persistently tracks the same object.

### 4.3. Component Ablation

To evaluate the effectiveness of the method’s design, we ablate the contributions of the proposed motion model and association method in LUKF-Track on the validation sets of MOT17 and DanceTrack in Table 2. As the data association makes the appearance information of objects an option in the first-round association, we form six trackers using different combinations of a motion model, appearance model, and data association. The two baseline trackers adopt a KF and UKF as motion models and a regular IoU calculation as the matching strategy. The fifth and sixth trackers are the two implementations of LUKF-Track. For a fair comparison, all trackers apply the same object detection, which is followed by that of ByteTrack. In the table, “**✓**” indicates that a proposed module is adopted in a tracker; “Fixed” indicates that the matching strategy associates the motion and appearance information with two fixed weights of 0.8 and 0.2.

**Motion Model.** Comparing the first and secnd trackers, we find that the UKF is a better motion model than the standard KF. The comparison between the second and third trackers shows that the performance gains from the proposed motion model are significant on both datasets for all metrics, which validates the effectiveness of modeling the dynamics and states of an object on the Lie group manifold. We also observe that the improvements to metrics are more significant on the DanceTrack dataset, where the motions of dancers are more complicated, and the interaction is heavier.

**Association Method.** Comparing the fourth with the fifth tracker, which both use motion and appearance information as the association metric, we find that the proposed two-round data association generally enhances the metrics of HOTA (i.e., from 68.6 to 71.5), which largely reflects the association performance.

**The Usage of Appearance Information.** The comparison between the fifth and sixth trackers shows that the effectiveness of appearance information is trivial and only presents notable improvements on the MOT17 dataset, in which the motion of objects is slower and the appearances of objects are more heterogeneous. It is worth noting that the three metrics are even reduced after adding the appearance model for DanceTrack; this could be explained by the negative effect caused by the homogeneous appearances of dancers.

## 5. Conclusions

We analyze popular MOT trackers and identify two major challenges in MOT tasks: missing association due to recognizing an object as background and false prediction caused by the predominant utilization of linear motion models and the insufficient discriminability of object appearance representations. Targeting these challenges, we propose a simple yet generic MOT method with a UKF on the Lie group named LUKF-Track. The method makes full use of detection boxes from high scores to low ones in a two-round data association and matches objects across frames by using a motion model where the propagation and prediction of object states are formulated by a UKF on the Lie group. In the experiments on three public MOT datasets, LUKF-Track outperforms other MOT trackers for comparison. The gain is especially significant for scenarios of severe occlusion and nonlinear object motion. We hope the high accuracy and simplicity of LUKF-Track can make it attractive in real applications.

## Figures and Tables

**Table 1:** Results of different tracking methods on different datasets.

Trackers	Similarity Measurement	MOTA	HOTA	IDF1
*MOT17-test*				
SORT	Motion	43.1	34.0	39.8
ByteTrack	Motion	**80.3**	63.1	77.3
OC-SORT	Motion	78.0	63.2	77.5
FineTrack	Appearance	80.0	**64.3**	79.5
QDTrack	Appearance	77.2	58.8	72.2
DeepSORT	Motion+Appearance	78.0	61.2	74.5
StrongSORT	Motion+Appearance	78.3	63.5	78.5
LUKF-Track (M+A)	Motion+Appearance	78.5	63.7	**79.6**
LUKF-Track (M)	Motion	78.2	63.7	79.5
*MOT20-test*				
SORT	Motion	42.7	36.1	45.1
ByteTrack	Motion	77.8	61.3	75.2
OC-SORT	Motion	75.5	62.1	75.9
FineTrack	Appearance	77.9	**63.6**	79.0
QDTrack	Appearance	74.7	60.0	73.8
DeepSORT	Motion+Appearance	71.8	57.1	69.6
StrongSORT	Motion+Appearance	72.2	61.5	75.9
LUKF-Track (M+A)	Motion+Appearance	**78.1**	61.9	**79.2**
LUKF-Track (M)	Motion	77.8	62.2	79.1
*DanceTrack-test*				
SORT	Motion	**91.8**	47.9	50.8
ByteTrack	Motion	89.5	47.3	52.5
OC-SORT	Motion	89.6	54.6	54.6
FineTrack	Appearance	89.9	52.7	**59.8**
QDTrack	Appearance	87.7	54.2	50.4
DeepSORT	Motion+Appearance	87.8	45.6	47.9
StrongSORT	Motion+Appearance	91.1	**55.6**	55.2
LUKF-Track (M+A)	Motion+Appearance	90.8	49.4	55.3
LUKF-Track (M)	Motion	91.3	49.8	56.1

**Table 2:** Component Ablation on MOT17-val and DanceTrack-val.

	Components			MOT17-val			DanceTrack-val		
	Mot	App	Ass	MOTA	HOTA	IDF1	MOTA	HOTA	IDF1
1	KF	×	IoU	79.8	62.9	72.5	78.2	40.6	45.0
2	UKF	×	IoU	80.6	63.3	72.9	79.1	39.8	45.2
3	**✓**	×	IoU	82.2	66.8	77.3	87.4	46.7	51.6
4	**✓**	**✓**	Fixed	85.8	68.6	82.1	87.5	45.9	50.4
5	**✓**	**✓**	**✓**	86.1	71.5	82.5	87.9	48.5	52.3
6	**✓**	×	**✓**	86.0	71.7	82.8	88.1	49.6	52.9

## Data Availability

The data presented in this study are available on request from the corresponding author.
